# Functionalization of Enzymatically Treated Apple Pomace from Juice Production by Extrusion Processing

**DOI:** 10.3390/foods10030485

**Published:** 2021-02-24

**Authors:** Vera Schmid, Antje Trabert, Judith (Schäfer) Keller, Mirko Bunzel, Heike P. Karbstein, M. Azad Emin

**Affiliations:** 1 Institute of Process Engineering in Life Sciences, Chair of Food Process Engineering, Karlsruhe Institute of Technology (KIT), 76131 Karlsruhe, Germany; vera.schmid@kit.edu (V.S.); heike.karbstein@kit.edu (H.P.K.); 2 Institute of Applied Biosciences, Department of Food Chemistry and Phytochemistry, Karlsruhe Institute of Technology (KIT), 76131 Karlsruhe, Germany; antje.trabert@kit.edu (A.T.); judith.schaefer@kit.edu (J.K.); mirko.bunzel@kit.edu (M.B.)

**Keywords:** plant cell walls, by-products, dietary fiber, non-starch polysaccharides, upcycling, valorization, rheological properties, viscosity

## Abstract

Food by-products can be used as natural and sustainable food ingredients. However, a modification is needed to improve the technofunctional properties according to the specific needs of designated applications. A lab-scale twin-screw extruder was used to process enzymatically treated apple pomace from commercial fruit juice production. To vary the range of the thermomechanical treatment, various screw speeds (200, 600, 1000 min^−1^), and screw configurations were applied to the raw material. Detailed chemical and functional analyses were performed to develop a comprehensive understanding of the impact of the extrusion processing on apple pomace composition and technofunctional properties as well as structures of individual polymers. Extrusion at moderate thermomechanical conditions increased the water absorption, swelling, and viscosity of the material. An increase in thermomechanical stress resulted in a higher water solubility index, but negatively affected the water absorption index, viscosity, and swelling. Scanning electron microscopy showed an extrusion-processing-related disruption of the cell wall. Dietary fiber analysis revealed an increase of soluble dietary fiber from 12.6 to 17.2 g/100 g dry matter at maximum thermo-mechanical treatment. Dietary fiber polysaccharide analysis demonstrated compositional changes, mainly in the insoluble dietary fiber fraction. In short, pectin polysaccharides seem to be susceptible to thermo-mechanical stress, especially arabinans as neutral side chains of rhamnogalacturonan I.

## 1. Introduction

Clarified apple juice is one of the most-consumed juices in the world. Enzyme technology is often used to increase the juice yield and for juice clarification [1,2,3]. Apple pomace remains as major by-product of juice processing. As only a small portion is used for pectin extraction or as animal feed [4], tons of apple pomace are disposed of as waste [5]. To date, there is no efficient method for the conversion of apple pomace into high-value products; thus, apple pomace has very little to no economic value. At the same time, there is a growing demand of consumers for healthy and sustainable foods [6,7].

Apple pomace is a source of potentially healthy nutrients. Being rich in dietary fiber (Total Dietary Fiber (TDF): 60–90 g/100 g dm) and phytochemicals such as phloridzin, chlorogenic acid, and quercetin glycosides [4,5,8,9,10], it has the potential to be used as a valuable food ingredient. However, it shows poor technofunctional properties, e.g., low water solubility and adsorption, as well as low or no thickening and gelling properties, limiting its industrial applications in food products [5,8,11,12].

Previous studies showed that this limitation can be overcome by modifying the cell wall structure, leading to a significant improvement of the technofunctional properties [6]. Chemical, enzymatic, thermal, and/or mechanical treatment can modify the technofunctional properties. In this regard, extrusion processing is a promising technology. It is a continuous, highly versatile process, in which raw materials are heated and sheared simultaneously [13,14].

Hwang et al. showed that extrusion processing of apple pomace resulted in an increase of soluble dietary fibers (SDF) from 8.2 to 18.2% and a decrease in insoluble dietary fiber (IDF) from 42.4 to 29.5%, whereas its water holding capacity (~4 mL·g^−1^) did not change at low thermomechanical treatment [15]. Liu et al. measured an increase in water solubility from 46 to 56.5 g·100 g^−1^ after the extrusion processing of apple pomace [16]. Also, an improvement in water-based pectin extraction after extrusion processing twice was observed [17]. The feasibility of using extrusion processing to modify apple pomace that has not been enzymatically treated during juice production was shown in our previous study. The results showed that the water solubility was increased from 5.2 to 33.0% and the water holding capacity of apple pomace from 7.3 to 21.4. Also, the viscosity of apple pomace water dispersions was (positively) affected and was increased from 5 up to 396 Pa·s [18]. Dietary fiber and structural analyses confirmed the observations of Hwang et al. [15] that thermo-mechanical treatment results in a decrease in IDF along with an increase in SDF. Arabinose and galacturonic acid containing polysaccharides (pectins) of the IDF fraction were partially degraded [18].

In this study, enzymatically treated apple pomace, as it is the major by-product of apple juice production, was used. The enzymatically treated pomace differs from untreated apple pomace as the cell wall polymers are partially modified during the enzymatic treatment. This is expected to affect the modification of technofunctional properties by extrusion processing. Thus, the main focus of this study was to investigate the impact of extrusion processing on the chemical structure and technofunctional properties of the already enzymatically modified apple pomace. Therefore, the change in the structure and technofunctional properties of enzymatically treated apple pomace was analyzed as a function of thermomechanical treatment during extrusion processing, which was varied to a great extent by varying the screw configuration, screw speed, and water content.

## 2. Materials and Methods

### 2.1. Materials, Chemicals, and Reagents

Conventional apple pomace, which was enzymatically treated during juice processing, was received from J. Rettenmaier & Söhne GmbH + Co KG (Rosenberg, Germany). The residual moisture content was 2.8 ± 0.3% (*w*/*w*) (determined by Karl Fischer titration (Titroline alpha, Schott Instruments GmbH, Mainz, Germany). According to the supplier, the dietary fiber content was 57.2 and the protein content 4.0 g per 100 g dm (dry matter). Thermostable α-amylase Termamyl 120 L (EC 3.2.1.1, from *Bacillus licheniformis*, 120 KNU·g^−1^), amyloglucosidase AMG 300 L (EC 3.2.1.3, from *Aspergillus niger*, 300 AGU·g^−1^), and protease Alcalase 2.5 L (EC 3.4.21.62, from *Bacillus licheniformis*, 2.5 AU·g^−1^ from Novozymes (Bagsværd, Denmark) were used to isolate dietary fiber on a preparative scale. Thermostable α-amylase (EC 3.2.11, from *Bacillus licheniformis*, 3000 U·mL^−1^), amyloglucosidase (EC 3.2.1.3 from *Aspergillus niger*), and protease (EC 3.4.21.14 from *Bacillus licheniformis*) (used for the analytical dietary fiber approach; the first two enzymes were also used to analyze starch contents), as well as endo-galactanase (EC 3.2.1.89 from *Aspergillus niger*) and endo-arabinanase (EC 3.2.1.99 from *Aspergillus niger*) were from Megazymes (Bray, Ireland). 

### 2.2. Extrusion Trials

**Figure 1:**
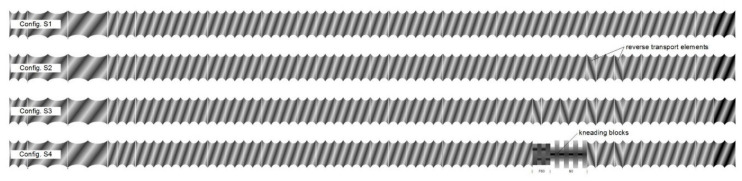
Schematic illustration of all screw configurations used. S1: only transport elements, S2 and S3: two and four reverse elements, S4: two reverse elements and a kneading block.

Extrusion trials were conducted using a co-rotating Thermo Scientific Process 11 hygenic twin-screw extruder (Thermo Fisher Scientific, Karlsruhe, Germany) with a length-to-diameter (L/D) ratio of 40. The barrel of the extruder consisted of eight sections, which could be heated and cooled separately. The barrel temperatures were adjusted to T_barrel1_ = 40 °C, T_barrel2_ = 80 °C, T_barrel3_ = 100 °C, and T_barrel5–8_ = 120 °C. The die adapter including the die (2 mm) was also heated to 120 °C. The apple pomace (8 kg·h^−1^) was fed into the first sections; water (2 kg·h^−1^) was added in the second section. Extrusion experiments were performed applying screw speeds of 200, 600, and 1000 min^−1^. The screw configurations are shown in Figure 1. From configuration S1 to S4, the thermomechanical treatment on the material increases, by the implementation of two reverse elements (S2), four reverse elements (S3), and two reverse elements plus a kneading block (S4). 

The melt temperature T_M_ was measured in the die adapter. The specific mechanical energy input (*SME*) was calculated according to the following equation (1):

(1) $$SME\;\left(\mathrm{Wh}\cdot {\mathrm{kg}}^{-1}\right)=\frac{\frac{n}{n_{max}}\times \frac{M_{d}-\;M_{d,\;unload}}{100}}{\dot{m}}\;\times P_{max}$$

where *n* and *n_max_* are the actual and maximum screw speeds (1000 min^−1^); *M_d_* and *M_d,unload_* are the actual and idle torques (%); $\dot{m}$ represents the total mass flow (1 kg·h^−1^) and *P_max_* the maximum engine power (1.5 kW). The residence time distribution (RTD) was measured by adding 0.1 g of TiO_2_ tracer to the barrel. By videography, 30 pictures per minute were taken and the color changes (L* value) were analyzed using MATLAB. RTD trails were performed in triplicate. 

The obtained extruded samples were vacuum packed and stored in the dark at room temperature.

### 2.3. Compositional and Structural Analysis

For all of the structural and technofunctional measurements, raw and extruded apple pomace was milled for 30 s (coffee mill M55, Petra Electric, Jettingen-Scheppach, Germany) and sieved (140 < x < 280 µm). The particle fraction was dried at 25 °C and 8 mbar in a vacuum dryer (Heraeus, Hanau, Germany) to constant weight.

All data are given as the mean ± standard deviation (*n* = 3). All data related to isolated dietary fiber (molecular weight distribution, polysaccharide composition and interunit linkage, arabinan and galacatan profiling, degree of (pectin) esterification) are presented as the mean ± range/2 (*n* = 2). Statistical analyses were performed for all data, except for data related to isolated dietary fiber, by using the software OriginPro 2020 9.7.0.188. Differences among varietier were tested for statistical significance using ANOVA, followed by a Tukey test (α = 0.005). 

**Free mono- and disaccharides.** Free mono- and disaccharides were analyzed after aqueous extraction (200 mg/10 mL water). The extraction process was accelerated by consecutive vortexing and sonication (temperature <30 °C), followed by centrifugation (10 min, 4500 rpm). This process was performed four times using fresh water after each centrifugation. 

The resulting supernatants were combined, and an aliquot was filtered through a PTFE membrane filter (45 µm). Ethanol (four times the volume of the aqueous aliquot) was added to precipitate residual polymeric material. The supernatant was evaporated, the residue was redissolved in water, and D-fucose (200 µM) was added as an internal standard. Chromatographic analysis was performed using high-performance anion-exchange chromatography with pulsed amperometric detection (HPAEC-PAD) (Thermo Fisher Scientific, Waltham, MA, USA) as described previously [18,19]. 

**Starch content.** A suspension of the sample (1 g) in 0.08 M phosphate buffer (50 mL, pH 6.0) was treated for 15 min at 60 °C. Enzymatic digestion (thermostable α‑amylase (3000 U·mL^−1^, 92 °C, 15 min) and amyloglucosidase (36,000 U·g^−1^, pH 4.5, 60 °C, 15 min) was applied to degrade the starch into glucose. Carrez clarification was performed (1 mL of Carrez I solution (150 g·L^−1^ K_4_[Fe(CN)_6_]·3H_2_O) and 1 mL of Carrez II solution (300 g·L^−1^ ZnSO_4_·7H_2_O)), and the pH was adjusted between 7.5 and 8.0. After centrifugation (5 min, 4500 rpm) and volume adjusting, the solution was diluted, and D-fucose (200 µM) was added as an internal standard. The starch content was determined as the sum of anhydroglucose, quantified using HPAEC-PAD (Thermo Fisher Scientific, Waltham, MA, USA) as described previously [18,19]. 

**Dietary fiber analysis.** The IDF, SDF, and low-molecular-weight soluble dietary fiber (LMW-SDF) were determined by a combination of the methods of AOAC 985.29 [20] and AOAC 2009.01 [21] in order to exclude resistant starch but capture ethanol soluble fiber as also described in [18]. After enzymatic digestion with thermostable α-amylase, amyloglucosidase, and protease, IDF and SDF were determined gravimetrically. Fiber contents were corrected for ash and residual protein (by Kjeldahl analysis) [22]. LMW-SDF was analyzed from the filtrate of SDF. The filtrate was evaporated, desalted, and redissolved in water. The aqueous solution was analyzed for its LMW-SDF content by high performance liquid chromatography (HPLC) (Hitachi, Merck, Darmstadt, Germany) with refractive index (RI) detection (Knauer, Berlin, Germany) using two size exclusion columns (TSKgel G2500PWxl, 300 mm × 7.8 mm, particle size 7 µm, Tosoh, Tokyo, Japan) [18]. 

**Dietary fiber isolation.** The IDF and SDF were isolated on a preparative scale according to the principle of AOAC 985.29 (incubation with thermostable α‑amylase, amyloglucosidase, and protease) as detailed by Bunzel et al. [23]. 

**Molecular weight distribution.** SDF (2 g·L^−1^) was dissolved in 50 mM sodium nitrate for 24 h at 40 °C. The molecular weight distribution of SDF was analyzed by HPLC-RI (Hitachi, Merck, Darmstadt, Germany) using a guard column (TosohTSK-gel PWxl 40 mm × 6.0 mm, particle size 12 µM) and two size exclusion columns in series (TosohTSK-gel G6000PWxl 300 mm × 7.8 mm, particle size 13 µM; TSK-gel G4000PWxl 300 mm × 7.8 mm, particle size 10 µM) with 50 mM sodium nitrate as eluent, a flow rate of 0.5 mL·min^−1^ and a temperature of 50 °C [18]. 

**Polysaccharide composition.** The monosaccharide composition of IDF was determined after sulfuric acid hydrolysis. SDF was depolymerized using methanolysis followed by TFA hydrolysis. Liberated monosaccharides were analyzed by HPAEC-PAD (Thermo Fisher Scientific, Waltham, MA, USA) [19]. To determine the composition of LMW-SDF, a preparative separation of mono- and disaccharides by HPLC (Hitachi, Merck, Darmstadt, Germany) with RI detection (Knauer, Berlin, Germany) was necessary as detailed in Schmid et al. [18]: The monosaccharide composition of extracted LWW-SDF was analyzed by HPAEC-PAD (Thermo Fisher Scientific, Waltham, MA, USA) after TFA hydrolysis [18].

**Analysis of polysaccharide interunit linkages.** To analyze the glyosidic linkages between the monosaccharides in the SDF and IDF polymers, methylation analysis was performed according to Gniechwitz et al. [24]. Fiber was swollen in dimethyl sulfoxide and treated twice with methyl iodide and NaOH. Methylated polysaccharides were extracted into dichloromethane, hydrolyzed with TFA (2 M, 121 °C) and reduced using sodium borodeuteride in aqueous ammonia. Acetylation was performed with acetic anhydride and 1‑methylimidazole. Obtained partially methylated alditol acetates were determined semi-quantitatively with gas chromatography-mass spectrometry (GC-MS) and GC with flame ionization detector (FID) using the molar response factors according to Sweet et al. [25]. 

**Arabinan and galactan screening.** Arabinan and galactan oligosaccharides were liberated from the neutral side chains of pectin by using endo-arabinanase and endo-galactanase and were determined semi-quantitatively using HPAEC-PAD (Thermo Fisher Scientific, Waltham, MA, USA) as described by Wefers and Bunzel [26]. 

**Degree of (pectin) esterification.** Methanol that was released from 15 mg of sample material after a 2 h deesterification step with sonication (2 M NaOH in D_2_O) was analyzed by ^1^H-NMR spectroscopy. 3-(Trimethylsilyl) propionic-2,2,3,3-*d*_4_ acid sodium salt solution in D_2_O (0.2 mg·mL^−1^) was added as an internal standard. A standard Bruker ^1^H‑NMR pulse program (zg30) was used: 65536 data points, acquisition time 3.28 s, relaxation delay (D1) 35 s [18]. Polymer-bound galacturonic acid was analyzed photometrically according to the method of Blumenkrantz et al. [27]. 

### 2.4. Scanning Electron Microscope

The samples were fixed with platinum (Pt). A LEO 1530 (Carl Zeiss, Oberkochen, Germany) at high vacuum was used during scanning electron microscopy (SEM) to observe the particles. All images were taken at an operating voltage of 5 kV.

### 2.5. Water Solubility Index (WSI) and Water Absorption Index (WAI)

The WSI and WAI were determined in triplicate according to Anderson et al. [28] as described detailed in Schmid et al. [18]. In brief, milled, sieved, and dried apple pomace (0.5 g) was added to 19.5 g of demineralized water. Samples were mixed on a vortex mixer for 1 min, followed by 24 h on an orbital shaker. For the WSI, samples were centrifuged at 4600 × *g* for 50 min at 20 °C. No centrifugation step was performed for the WAI; instead, samples rested for 3 h. For both WSI and WAI supernatants were removed, dried (80 °C, 72 h), and weighed (*m*), just as the precipitates. The *WSI* and *WAI* were calculated according to the following equations:

(2) $$WSI=\;\frac{m_{supernatant,\;\;dried}}{m_{powder}}$$

(3) $$WAI=\;\frac{m_{precipitate,\;wet}-m_{precipitate,\;dried}}{m_{precipitate,\;dried}}$$

### 2.6. Rheological Measurements

Dispersions of raw or extruded apple pomace and water were made by mixing 1 g of ground, sieved, and dried sample with 10 mL of demineralized water. The dispersion was stirred on a magnetic mixer (200 min^−1^) for 10 min, sealed with parafilm and rested for 50 min. The rheological properties were determined by Anton Paar Rheometer MC 301 (Graz, Austria) using a parallel plate system (50 mm, smooth). The measurement gap was adjusted to 1.5 mm. After a rest of 90 s in the geometry, samples underwent oscillatory shear at 25 °C with an amplitude of 0.1% and a frequency of 1 Hz (within the linear viscoelastic region to avoid a destruction of a network). The shown complex viscosity η* is a mean value of the last nine measurement points of each measurement. The measurements were performed in triplicate. 

### 2.7. Swelling by Using a Light Microscope

The swelling of particles was determined by light microscopic (Eclipse LV100ND; Nikon, Tokyo, Japan) image processing. Demineralized water and sieved, dried pomace particles (140 < x < 280 µm) were placed on the microscope slide and observed for 100 min at room temperature. The growth of particle size was evaluated by using ImageJ. Experiments were performed in duplicate.

## 3. Results & Discussion

### 3.1. Influence of Extrusion Parameters on the Extent of Thermomechanical Treatment

**Figure 2:**
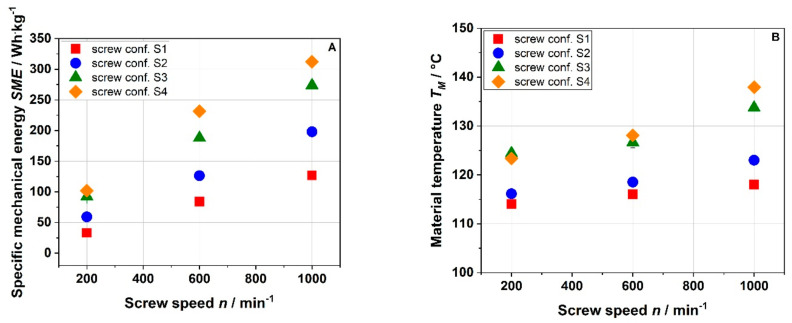
Specific mechanical energy input (SME) (**A**) and material temperature (T_M_) (**B**) for conventional apple pomace treated at various screw speed and screw configurations (S1: only transport elements, S2: two reverse elements, S3: four reverse elements, S4: two reverse elements and a kneading block) at 22% water content and a barrel temperature T_B_ of 120 °C.

To characterize the thermomechanical stresses applied to the raw material, the specific mechanical energy input (SME) and the material temperature (T_M_) were monitored. Figure 2 shows the influence of the screw speed on SME and T_M_ for the screw configurations used. 

In this study, the barrel temperature, water feed, and solid feed were kept constant; only the screw configuration and screw speed were varied. Increasing the screw speed from 200 to 1000 min^−1^ at a barrel temperature of 120 °C resulted in an increase in the SME for all screw configurations (Figure 2A). Screw configuration S1 showed the lowest SME, which increased by a factor of ~3.8 by increasing the screw speed from 200 to 1000 min^−1^. As expected, the SME increased with the number of reverse elements (S2 and S3). The highest SME values were observed for screw configuration S4, which included two reverse elements plus a kneading block. Reverse elements and kneading blocks restrict the flow of the raw material and intensify the mixing and shear, thus increasing the energy input (SME) [29].

SME and T_M_ (Figure 2B) are linked with each other as viscous dissipation results in temperature increase. The lowest T_M_ was observed for screw configuration S1, which consisted only of forward elements. The effect of the screw speed on the processing conditions was a function of the screw configuration used. Compared to a mild screw configuration (S1), the use of reverse elements as well as kneading blocks (S2–S4) led to a higher increase in the intensity of thermomechanical treatment with screw speed. This effect was more apparent for SME than T_M_. 

**Figure 3:**
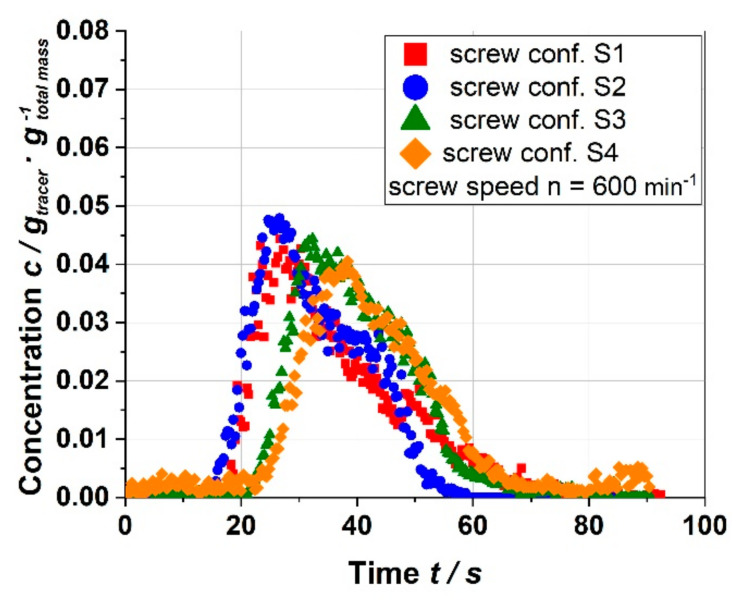
Residence time distribution for various screw configurations (screw speed: 600 min^−1^ and water content: 22%, barrel temperature: 120 °C; S1: only transport elements, S2: two reverse elements, S3: four reverse elements, S4: two reverse elements and a kneading block).

The residence time of the material in the extruder depends also on the screw configuration. At longer residence time, the material is exposed to the thermal stresses for a longer time. In addition to this, higher residence increases the probability that the material is exposed more often to the maximum thermal and mechanical stresses generated between the barrel wall and the tip of the screws [30]. Therefore, the chemical structures, and as a result, the technofunctional properties are potentially affected by the residence time. Figure 3 shows the residence time distribution (RTD) of all screw configurations used.

In general, the use of more restrictive elements is expected to result in longer residence time. Configurations S1 and S2, however, led to comparable RTDs within the range of 18 to 91 s and 17 to 65 s, and mean residence times of 37 and 35 s, respectively. 

In comparison, S3, with more screw elements, led to a significantly longer residence time with a range of 25 to 67 s and a mean residence time of 41 s.

The longest residence time was observed for S4, with the range of 27 to 69 s and a mean residence time of 44 s.

Overall, the results show that changing the screw speed and configuration led to a variation in thermomechanical treatment to a great extent with SME, T_M,_ and mean residence time in the ranges of 33 to 312 Wh·kg^−1^, 114 to 138 °C, and 33 to 45 s, respectively. 

### 3.2. Influence of Extrusion Conditions on the Structure and Composition of Conventional Apple Pomace

The impact of extrusion conditions on the structural characteristics of enzymatically treated apple pomace was examined by using screw configurations S1, S2, and S4. For each configuration, a barrel temperature of 120 °C, a screw speed of 600 min^−1^, and a water content of 22% were used. Because the focus of this study was on non-starch polysaccharides, results and additional methods for other apple pomace constituents (before and after extrusion) are deposited as Appendix (Method Appendix A; protein, ash: Table A1; free mono- and disaccharides: Table A2; starch: Table A3). Most importantly, contents of mono-/disaccharides and starch did not change after extrusion, independent of the screw configuration used. 

#### 3.2.1. Dietary Fiber Composition

The IDF and SDF contents of enzymatically treated apple pomace were affected by extrusion, as previously demonstrated [15,18]. The IDF slightly decreased, whereas the SDF increased with intensified thermo-mechanical treatment (Table 1). In contrast to apple pomace that has not been enzymatically treated during juice processing [18], the raw material used in this study already contained LMW-SDF. LMW-SDF generated from cell wall polysaccharides during juice extraction was potentially not fully extracted into the juice. However, correlations between LMW-SDF contents and thermomechanical treatment, residence time, or screw configuration were not observed. 

Dietary fiber contents of apple pomace were studied in the past [8,18,31] demonstrating that apple variety affects fiber contents. To the best of our knowledge, however, the fiber contents of enzymatically treated apple pomace have not yet been analyzed. Nevertheless, the values analyzed here are the range of published data for untreated apple pomace (IDF 33–67%, SDF 3–14%). 

#### 3.2.2. Polysaccharide Characterization: Molecular Weight Distribution of Soluble Dietary Fiber Polysaccharides

**Figure 4:**
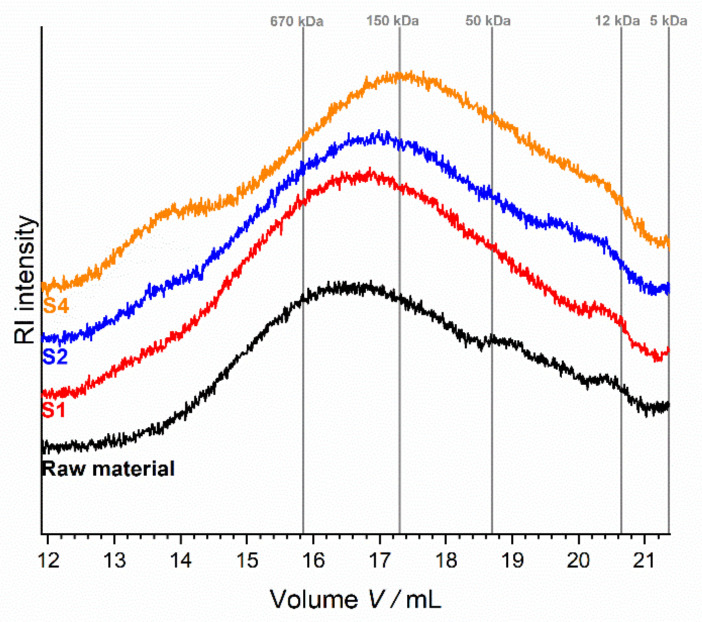
Molecular weight distribution of soluble dietary fiber (SDF) of enzymatically treated apple pomace (raw and extruded) as determined by using HPLC with refractive index (RI) detection. Dextrans of defined molecular weight were used for calibration (grey lines). S1: only transport elements, S2: two reverse elements, S4: two reverse elements and a kneading block.

The SDF data indicated that the structure of dietary fiber polysaccharides was mostly influenced by screw configuration S4 (highest thermo-mechanical stress). This is in line with data describing the molecular weight distribution (Figure 4). Only a slight broadening of the distribution was observed for SDF from samples treated with screw configurations S1 and S2, whereas broadening was more distinct for SDF from the sample treated with configuration S4. Interestingly, the SDF obtained from the sample treated with screw configuration S4 showed an additional peak (elution volume of 13.6 mL (> 670 kDa)). This might be due to the formation of high molecular melanoidins as end products of the Maillard reaction. Generally, these results are in line with our previous data for apple pomace that has not been treated during juice extraction [18].

#### 3.2.3. Polysaccharide Characterization: Monomer Composition and Interunit Linkages of Fiber Polysaccharides

The monosaccharide composition of fiber polysaccharides was determined after sulfuric acid hydrolysis (IDF) (Table 2) or after methanolysis (SDF) (Table 5), respectively. Sulfuric acid hydrolysis tends to underestimate uronic acids including galacturonic acid, whereas crystalline cellulose is not hydrolyzed using methanolysis. 

Cellulose, pectic polymers, and the hemicellulose xyloglucan have been previously described as dominant IDF polysaccharides from apples or apple pomace [18,32]. A comparable composition was found for enzymatically treated apple pomace: raw material IDF contained glucose (49.6 mol%) as the main monosaccharide (vs. 43.3% in IDF from untreated apple pomace [18]). Besides cellulose, glucose can also be released from the hemicellulose xyloglucan. Xylose (10.4 mol%) may stem from xyloglucans, but can also be released from xylans and xylogalacturonans. Arabinose (15.6 mol%) and galactose (9.1 mol%) are monomers of the neutral side chains of rhamnogalacturonan I. Galacturonic acid (8.2 mol%) and rhamnose (1.8 mol%) are pectic polysaccharide backbone constituents (homogalacturonan, rhamnogalacturonan I). Thus, up to 36 mol% of the liberated monomers (including fucose) can be preliminarily assigned to pectic polymers, less than the IDF of untreated apple pomace (43 mol%) [18]. Because pectins are supposed to be a major target of the enzymes used, the shift in the overall monomer composition can easily be explained. However, other factors (apple variety, storage of apples etc.) may also affect the composition of the pomace after juice extraction. 

Assignment of the liberated monosaccharides to individual polysaccharides can be (partially) performed by using methylation analysis data (analysis of interunit linkages). Results of the neutral pectic side-chain profiling approach provided additional information about the structure of IDF polysaccharides. Data of the glycosidic linkage analysis of IDF are given in Table 3. Glucose was mainly 1,4-linked, suggesting, as expected, large amounts of cellulose. Besides cellulose, the xyloglucan backbone contains 1,4-linked glucopyranose units, too. Because 1,4,6‑linked glucopyranose and terminal xylopyranose units were also identified, minor portions of 1,4-linked glucopyranose can be attributed to xyloglucans. 1,2-linked xylopyranose is a known structural element of xyloglucans. Only small amounts of 1,4-linked xylopyranose (3.8 mol%) and 1,4-linked mannopyranose (3.8 mol%) units were detected in enzymatically treated apple pomace IDF, suggesting that xylans and linear mannans are quantitatively less important. The partially methylated alditol acetates of arabinose suggest arabinans (1,5-linked arabinofuranose as linear backbone units) with branches in positions *O*-3 (primarily) and/or *O*-2. Glycosidic linkages of galactose units indicate a primarily linear structure.

**Figure A1:**
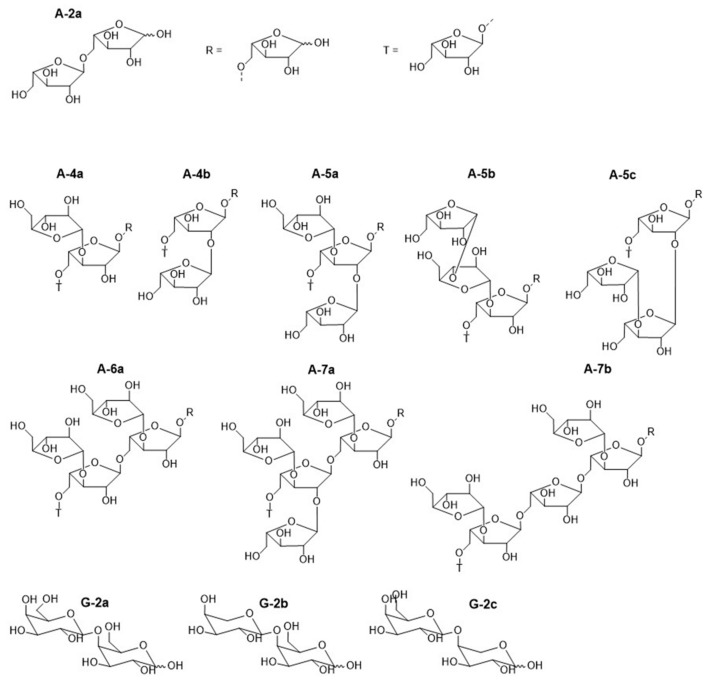
Arabinan and galactan oligosaccharides of IDF and SDF of enzymatically treated apple pomace (raw and extruded) after enzymatic cleavage by endo-arabinanase and endo-galactanase. IDF: insoluble dietary fiber, SDF: soluble dietary fiber, R: reducing end, T: terminal end.

Structures of the neutral pectic side chains were verified using a profiling approach published by Wefers and Bunzel [26] (Table 4). In addition to backbone oligosaccharides with branches in position *O*-3 (e.g., A-4a; Figure A1, Appendix B), *O*-2 (e.g., A-4b) or in both positions (e.g., A-5a) structural units that demonstrate dimeric side-chains (e.g., A-5b) were identified. Thus, the profiling approach verified methylation analysis data with regard to the predominance of branching in position *O*-3 over branching in position *O*-2 and branching in both positions. G-2a was the predominantly observed galactan oligosaccharide, a disaccharide representing 1,4-linked galactopyranose units of the galactan backbone (Table A4, Appendix B). Furthermore, structures that contain arabinopyranose units were identified: G-2c demonstrating internal 1,4-arabinopyranose and low amounts of G-2b demonstrating terminal arabinopyranose units. The presence of these galactan structural elements in apples was previously demonstrated by Wefers and Bunzel [32].

Monosaccharide composition data of IDF obtained from extruded samples revealed that increased thermo-mechanical stress results in decreased arabinose portions (down to 9.2 mol%) being consistent with data on extruded apple pomace that has not been enzyme treated [18]. This decrease is also reflected by methylation analysis data. Although there is no dramatic degradation of a single structural unit, it appears that extrusion-based changes affect slightly more branching positions, going along with decreased terminal arabinofuranose units. The decrease of 1,3,5- and 1,2,5-linked arabinofuranose units seems to be independent of the strength of the thermo-mechanical treatment. Only the reduction of terminal arabinofuranose units was more distinct when screw configuration S4 was used. Data of the arabinan profiling do not exactly match the methylation analysis data. Here, the portion of the *O*-3 branched oligosaccharide A-4a did not decrease; however, the portion of the more strongly branched oligosaccharide A-5b, which also contains the 1,3,5-linked arabinofuranose unit, decreased. The same applies to the oligosaccharides with backbone linkages in position *O*-2 (A-4b and A-5c, respectively), indicating that these slightly more complex side-chains are susceptible to extrusion-based degradation. More generally, the susceptibility of arabinans to thermomechanical stress has already been demonstrated for non-enzymatically treated apple pomace [18] and chokeberry pomace [33]. 

Besides arabinose, the portion of galacturonic acid appears to decrease with increasing thermo-mechanical stress (monosaccharide composition). This is accompanied by an increase in the portions of xylose and glucose. Overall, it can be recognized that treatments using screw configurations S1 and S2 result in comparable IDF polysaccharide modifications (based on monomer composition). As shown in Figure 2, configurations S1 and S2 result in similar T_M_, whereas configuration S4 generates a markedly higher T_M_. Furthermore, configuration S4 results in a much longer residence time as compared to configurations S1 and S2.

SDF polysaccharides consisted mostly of pectic polymers (about 88 mol%, Table 5). Just as in IDF, the dominant neutral side chains were arabinans as indicated by a higher portion of arabinose (39.9 mol%) as compared to galactose (10.3 mol%). Methylation analysis data (Table A5, Appendix B) confirmed that SDF polysaccharides mostly consist of arabinan rich pectins. Again, arabinan branching in position *O*-3 dominates over branching in position *O*-2, as also demonstrated by the results of the arabinan profiling approach (Table A6, Appendix B). Dimeric side-chains attached in position *O*-3 of the backbone (reflected by the oligosaccharide A-5b liberated in the arabinan profiling approach) were also found in appreciable portions in SDF arabinans. Xyloglucan-specific linkage types such as 1,4,6-linked glucopyranose units in combination with 1,2-linked xylopyranose units (Table A5, Appendix B) demonstrate the existence of xyloglucans in this fiber fraction, although in minor amounts only. 

Analysis of the extruded samples revealed that the galacturonic acid content in the SDF polysaccharides decreased. All other changes were rather minor; the amount of arabinose in the SDF polysaccharides does not appear to be largely affected by thermo-mechanical treatment. Methylation analysis failed to demonstrate major changes in the interunit linkages of the SDF polysaccharides before and after extrusion (Table A5, Appendix B). The only more pronounced and consistent difference is an increase in terminal arabinofuranose units. The arabinan (Table A6, Appendix B) and galactan profiling approaches (Table A4, Appendix B) did not show any further evidence of specific extrusion based structural changes of SDF polysaccharides. 

The LWD-SDF fractions of the raw material and the extruded samples mainly contain arabinose, glucose, and galacturonic acid. Although the arabinose portions appear to increase and the glucose portions appear to decrease with thermo-mechanical treatment, an unambiguous statement is difficult (Table A7, Appendix B). 

#### 3.2.4. Polysaccharide Characterization: Pectin Esterification

It is known that the degree of pectin esterification has an influence on the gel-forming properties of pectin. Therefore, the degree of polymer-bound galacturonic acid esterification was studied in the raw material and after extrusion. Yet, extrusion seems to have no distinct impact on the degree of esterification of enzymatically treated apple pomace. The IDF pectins of the raw material showed a degree of esterification of 18%. With the maximum thermomechanical treatment (screw configuration S4), the percentage was 15%. Only minor changes were also observed for the SDF pectins, where the portion increased from 23% (raw material) to 26% (screw configuration S4). Given the fact that these numbers are based on two separate analyses (analysis of polymer-bound galacturonic acid and analysis of methanol after esterification) the analyzed esterification degrees do not indicate extrusion based modifications of this parameter.

#### 3.2.5. Influence of Process Conditions on Surface of Extrudate Particles

**Figure 5:**
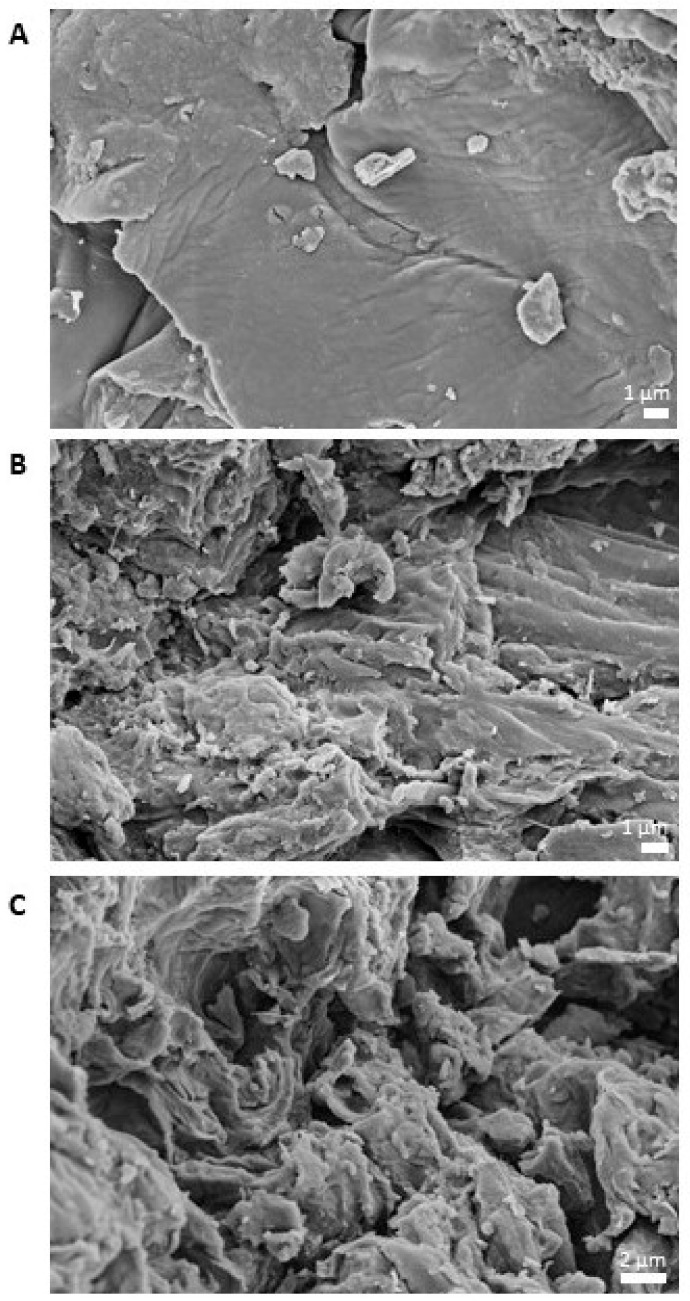
Scanning electron microscope images of conventional apple pomace surfaces. (**A**) Raw material. (**B**) Extruded, screw speed: 200 min^−1^, screw configuration S1, barrel temperature: 120 °C, water content: 22%. (**C**) Extruded, screw speed: 200 min^−1^, screw configuration S4, barrel temperature: 120 °C, water content: 22%.

Besides chemical structure, the macroscopic structure is also altered by extrusion processing. Figure 5 depicts scanning electron microscope (SEM) images of raw and extruded apple pomace.

Figure 5A shows the smooth, closed surface of the raw apple pomace, which was enzymatically treated during juice processing. Figure 5B–C show samples that were extruded using screw configuration S1 or S4, respectively. The images show that an application thermomechanical treatment results in a rougher surface, which becomes more prominent at higher treatment intensity (Figure 5C). 

### 3.3. Influence of Extrusion Conditions on the Functional Properties of Apple Pomace

#### 3.3.1. Water Solubility Index and Water Absorption Index

**Figure 6:**
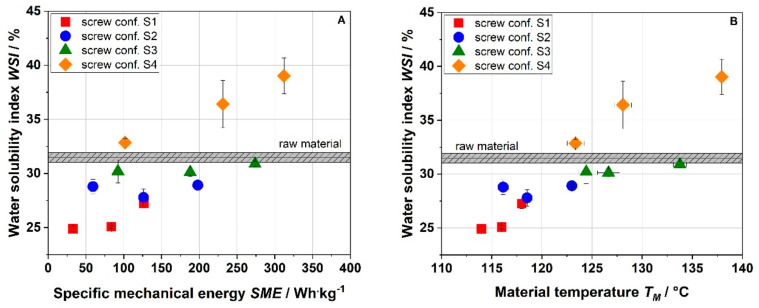
Effect of specific mechanical energy SME (**A**) and material temperature T_M_ (**B**) on water solubility index (WSI) of apple pomace for various screw configurations at a water content of 22%. S1: only transport elements, S2: two reverse elements, S3: four reverse elements, S4: two reverse elements and a kneading block.

The water solubility indices (WSI) of raw and extruded apple pomace are shown in Figure 6. The WSI of raw material was 31.5%. By extrusion processing, the WSI can be increased by 14%. At milder process conditions (S1), however, the WSI was lower than the WSI of the raw material. An increase in the thermomechanical treatment by an increase in screw speed and change in screw configuration (S1 < S2 < S3 < S4) led to higher WSI values. The correlation between the SME and WSI was also observed by Hwang et al. [15], however, only for one screw configuration. Application of the most intensive thermomechanical treatment (S4) resulted in the highest WSI, as well as the highest amount of SDF (Table 1), which is probably due to an intensive degradation of cell wall polymers. These results show that, in addition to the SME and T_M_, the screw configuration had a significant effect, which could be related to the change in the residence time (Figure 3) leading to longer and more frequent exposure of the material to the local maximum stresses along the extruder screws [34]. 

**Figure 7:**
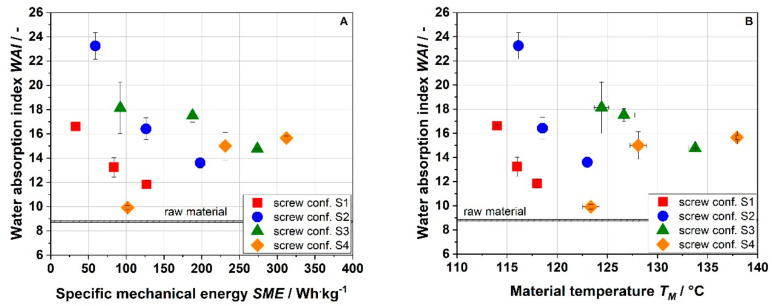
Effect of specific mechanical energy SME (**A**) and material temperature T_M_ (**B**) on water absorption index (WAI) of apple pomace for various screw configurations at a water content of 22%. S1: only transport elements, S2: two reverse elements, S3: four reverse elements, S4: two reverse elements and a kneading block.

Figure 7 shows the water absorption indices (WAI) of raw and extruded apple pomace. The WAI of the raw material was 8.8. Depending on the process conditions, the WAI was increased up to 23.3. For milder process conditions (S1) and moderate process conditions (S2, S3), the WAI decreased with the SME and T_M_. In contrast, at intensive process conditions (S4), the WAI increased with the SME and T_M_. Scanning electron microscope images of the surface of the raw and extruded apple pomace particles showed that by extrusion processing the surface was broken up (Figure 5). It can be assumed that by this alteration more water can infiltrate and be absorbed. Intensification of thermomechanical stress may disrupt the macromolecular structure to an extent that water absorption decreases.

#### 3.3.2. Swelling

**Figure 8:**
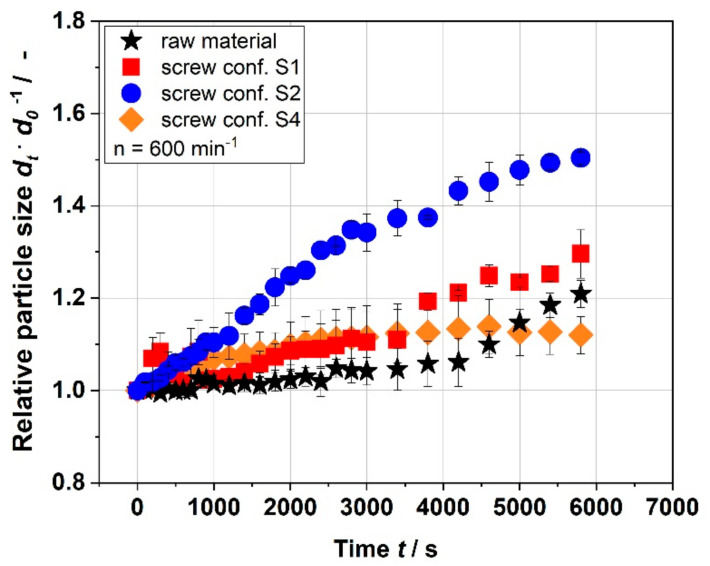
Change of size of raw material and various extruded samples by microscopic image analysis for a time period of almost 1 h 40 min. The relative particle size is the ratio between the area occupied by a particle at time t (A_t_) and the area occupied by the particle at the initial time (A_0_). S1: only transport elements, S2: two reverse elements, S4: two reverse elements and a kneading block).

Figure 8 shows the change in the size of raw and extruded apple pomace particles swelling in water. Samples, which were characterized structurally in detail, were chosen for the swelling investigations.

**Figure 9:**
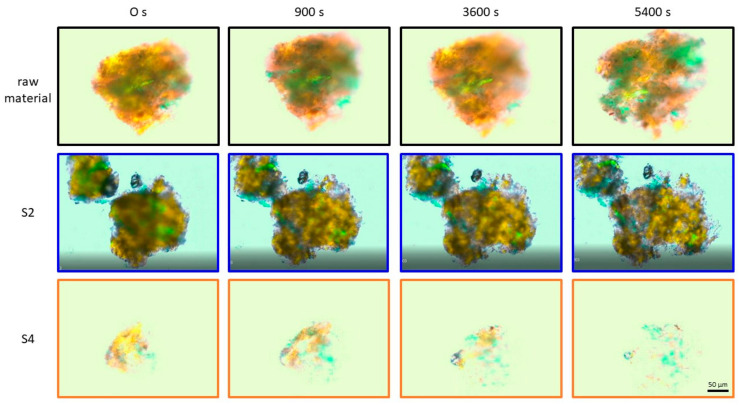
Microscope pictures of raw material, screw configuration S2 and S4 at 600 min^−1^ at 0, 900, 3600, 5400 s. S2: two reverse elements, S4: two reverse elements and a kneading block).

The results in Figure 8 show that the size of the raw material particles did not change for almost 4500 s. Afterwards, the particles of the raw material started to grow. In comparison, the extruded samples resulted in an increase in particle size right from the beginning. For moderate process conditions (S2), swelling of the apple pomace particles was most pronounced. The milder (S1) and most intense thermomechanical treatment (S4) had a smaller impact on particle size growth. Comparing the results with the WAI data (Figure 7), it can be concluded that the samples that swell to a larger extend also absorb more water, as expected. Figure 9 shows that the particles extruded at the most intense conditions (S4) are the smallest and least compact, which is in accordance with the surface structure depicted by SEM images in Figure 5. 

These results might suggest that the extrusion processing leads to more porous cell wall materials that could absorb more water than raw material, whereas excessive treatment could lead to fragmentation and decrease in this porous structure necessary for the water absorption and swelling. From a structural point of view (Table 1, Table 2, Table 3, Table 4 and Table 5), samples that were extruded using screw configurations S1 and S2 were comparable, whereas the sample that was extruded applying configuration S4 showed more distinct differences in structure, which is in accordance with these findings. Nevertheless, more investigations are necessary to draw a strong conclusion.

#### 3.3.3. Viscosity

**Figure 10:**
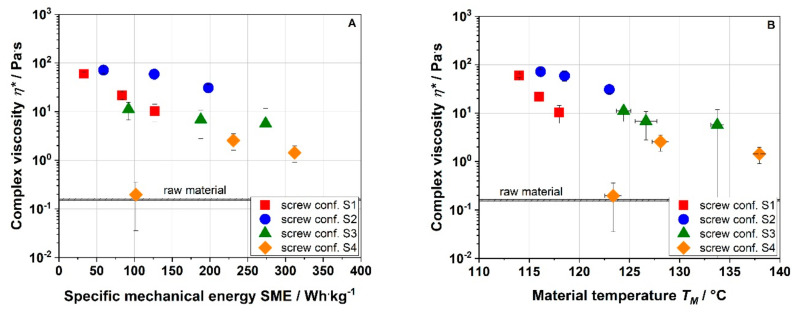
Effect of specific mechanical energy SME (**A**) and material temperature T_M_ (**B**) on complex viscosity (η*) of apple pomace for various screw configurations at water content of 22%. S1: only transport elements, S2: two reverse elements, S3: four reverse elements, S4: two reverse elements and a kneading block.

For the application of apple pomace as a thickener and stabilizer in food matrices, it is essential to know the rheological behavior. The complex viscosities of apple pomace-water dispersions are depicted in Figure 10.

The complex viscosity of the raw material was 0.16 Pa·s. Both low and moderate thermomechanical treatment (S1 and S2) resulted in an immense increase in complex viscosity up to max. 71.4 Pa·s (S2, 200 min^−1^). With increasing SME and T_M_, the complex viscosity decreased for screw configurations S1–S3. In contrast, for the screw configuration S4, which represents the highest thermomechanical treatment, the values of complex viscosity increased from 0.15 to 2.55 to 1.43 Pa·s with increasing SME and T_M_. These results are similar to the WAI results (Figure 7), suggesting that the water absorption and swelling (Figure 8 and Figure 9) due to macromolecular cell wall alterations play an important role in the thickening behavior of the extruded samples. Thus it is all about swelling not the gelling of individual components as assumed [11,35,36].

## 4. Conclusions 

This study investigates the functionalization of enzymatically treated apple pomace from commercial fruit juice production by extrusion processing. The results showed that the total DF content changed only slightly by varying the intensity of thermomechanical treatment, whereas the amount of SDF increased. The results may indicate a specific degradation reaction into the pectin polysaccharides. In detail, a decrease of arabinose and galacturonic acid in the IDF and a decrease of galacturonic acid in the SDF could be determined. The extrusion of the samples led to the modification of water solubility, water absorption, and viscosity. 

Depending on the conditions, the WSI could be varied in the range of 24.9 to 39.0%, whereas the WAI was highest for moderate thermomechanical treatment (23.3) and lowest for intensive thermomechanical treatment (9.9), which is slightly higher than raw material (8.8). The viscosity of apple pomace–water dispersion was increased from 0.2 up to 71.4 Pa·s. The analysis of the macrostructure suggests that the extrusion leads to a more porous structure of apple pomace particles, which is important for the swelling of the particles and thus for water absorption and viscosity, respectively. 

The functional properties showed no apparent correlation with SME and TM. For more mechanistic analysis, the analysis of the local extrusion conditions and the application of defined treatments should be considered in further studies. Nevertheless, the modification of enzymatically treated apple pomace makes it suitable as a potential natural thickener in dessert and dairy products or as a filler in bread and sausage products.

## Figures and Tables

**Table 1:** Dietary fiber contents (g/100 g dm*;* mean value ± standard deviation, *n* = 3) of enzymatically treated apple pomace (raw and extruded). IDF: insoluble dietary fiber, SDF: soluble dietary fiber, LMW-SDF: low-molecular weight soluble dietary fiber. S1: only transport elements, S2: two reverse elements, S4: two reverse elements and a kneading block.

	Raw Material	S1	S2	S4
Total content ^a^	52.7 ± 2.6 ^A,B^	56.4 ± 2.4 ^A,C^	52.3 ± 3.1 ^B^	57.1 ± 3.1 ^C^
IDF ^a^	38.3 ± 1.0 ^A,B^	40.4 ± 0.7 ^A^	37.6 ±1.3 ^A,B^	36.7 ±1.3 ^B^
SDF ^a^	12.6 ± 1.1 ^B^	12.9 ± 0.5 ^B^	13.1 ± 1.5 ^B^	17.2 ±1.3 ^A^
LMW-SDF ^a^	1.8 ± 0.5 ^A^	3.1 ± 1.2 ^A^	1.6 ± 0.3 ^A^	3.2 ± 0.4 ^A^

^a^ Mean values within a row that are marked with different letters differ significantly (*p* < 0.05).

**Table 2:** Monosaccharide composition (mol%) of insoluble dietary fiber (IDF) of enzymatically treated apple pomace (raw and extruded) after sulfuric acid hydrolysis (mean value ± range/2, *n* = 2) Fuc: fucose, Rha: rhamnose, Ara: arabinose, Gal: galactose, Glc: glucose, Xyl: xylose, Man: mannose, GalA: galacturonic acid; GlcA: glucuronic acid, S1: only transport elements, S2: two reverse elements, S4: two reverse elements and a kneading block, bdl: below detection limit.

	Raw Material	S1	S2	S4
Fuc	1.1 ± 0.02	1.2 ± 0.04	1.2 ± 0.1	1.3 ± 0.01
Rha	1.8 ± 0.03	1.7 ± 0.003	1.6 ± 0.001	1.4 ± 0.02
Ara	15.6 ± 0.01	13.1 ± 0.1	12.5 ± 0.03	9.2 ± 0.04
Gal	9.1 ± 0.2	8.2 ± 0.01	8.3 ± 0.03	8.0 ± 0.1
Glc	49.6 ± 0.01	51.1 ± 0.1	51.8 ± 0.04	56.4 ± 0.1
Xyl	10.4 ± 0.1	12.8 ± 0.3	13.2 ± 0.2	14.1 ± 0.2
Man	4.2 ± 0.1	4.1 ± 0.1	4.4 ± 0.01	4.6 ± 0.01
GalA	8.2 ± 0.1	7.7 ± 0.1	6.9 ± 0.2	5.0 ± 0.1
GlcA	^bdl^	^bdl^	^bdl^	^bdl^

**Table 3:** Glyosidic linkage of insoluble dietary fiber (IDF) of enzymatically treated apple pomace (raw and extruded) (mol%, mean value ± range/2, *n* = 2). t: terminal, p: pyranose, f: furanose, Rha: rhamnose, Ara: arabinose, Gal: galactose, Glc: glucose, Man: mannose, Xyl: xylose, S1: only transport elements, S2: two reverse elements, S4: two reverse elements and a kneading block.

	Raw Material	S1	S2	S4
1,2-Rha*p*	0.8 ± 0.1	1.0 ± 0.3	0.9 ± 0.01	0.9 ± 0.4
1,2,4-Rha*p*	0.4 ± 0.1	0.5 ± 0.1	0.9 ± 0.3	0.4 ± 0.1
∑ Rha	1.2 ± 0.1	1.5 ± 0.4	1.8 ± 0.3	1.2 ± 0.5
t-Ara*f*	8.6 ± 0.05	8.5 ± 1.4	7.1 ± 0.2	6.0 ± 0.03
t-Ara*p*	0.6 ± 0.01	0.5 ± 0.05	0.6 ± 0.04	0.6 ± 0.04
1,2-Ara*f*	0.5 ± 0.01	0.3 ± 0.02	0.5 ± 0.1	0.3 ± 0.04
1,3-Ara*f*	1.8 ± 0.05	1.5 ± 0.3	1.2 ± 0.05	1.4 ± 0.3
1,5-Ara*f*/1,4-Ara*p*	11.5 ± 0.03	8.5 ± 1.2	7.1 ± 0.5	9.4 ± 2.4
1,2,5-Ara*f*	1.7 ± 0.04	0.7 ± 0.2	0.6 ± 0.1	0.7 ± 0.02
1,3,5-Ara*f*	5.3 ± 0.02	3.0 ± 1.7	3.3 ± 0.4	3.3 ± 0.1
1,2,3,5-Ara*f*	7.5 ± 0.1	2.3 ± 0.3	2.6 ± 0.5	2.3 ± 0.02
∑ Ara	37.4 ± 0.3	25.4 ± 5.1	23.1 ± 1.9	23.9 ± 3.0
t-Gal*p*	3.7 ± 0.01	3.2 ± 0.04	2.5 ± 0.1	3.3 ± 1.0
1,4-Gal*p*	4.0 ± 0.03	3.6 ± 0.2	3.6 ± 0.1	3.7 ± 0.8
1,6-Gal*p*	0.6 ± 0.02	0.5 ± 0.03	0.5 ± 0.04	0.7 ± 0.2
1,4,6-Gal*p*	0.3 ± 0.004	0.3 ± 0.1	0.2 ± 0.02	0.3 ± 0.1
∑ Gal	8.6 ± 0.1	7.5 ± 0.3	6.7 ± 0.3	8.0 ± 2.0
t-Glc*p*	0.9 ± 0.1	0.9 ± 0.1	0.8 ± 0.01	0.9 ± 0.4
1,4-Glc*p*	24.9 ± 0.2	36.6 ± 4.2	41.3 ± 2.6	32.8 ± 3.1
1,4,6-Glc*p*	4.7 ± 0.04	7.8 ± 0.8	8.1 ± 0.7	7.0 ± 2.2
∑ Glc	30.5 ± 0.4	45.3 ± 5.0	50.1 ± 3.3	40.7 ± 5.7
t-Man*p*	0.3 ± 0.1	0.3 ± 0.1	0.2 ± 0.0005	0.2 ± 0.1
1,4-Man*p*	3.8 ± 0.04	3.2 ± 0.2	2.8 ± 0.5	4.2 ± 1.6
1,4,6-Man*p*	0.6 ± 0.02	0.5 ± 0.05	0.5 ± 0.1	0.7 ± 0.2
∑ Man	4.7 ± 0.2	4.0 ± 0.4	3.4 ± 0.6	5.2 ± 1.9
t-Xyl*p*	9.4 ± 0.05	8.9 ± 0.1	8.1 ± 2.8	10.3 ± 0.9
1,2-Xyl*p* ^a^	4.4 ± 0.02	3.5 ± 0.2	2.8 ± 0.2	4.6 ± 0.4
1,4-Xyl*p* ^a^	3.8 ± 0.01	4.0 ± 0.7	3.9 ± 0.3	6.1 ± 0.9
∑ Xyl	17.6 ± 0.1	16.4 ± 1.1	14.8 ± 1.2	20.9 ± 2.2

^a^ Coeluting, determined from the area ratio of the characteristic fragment ion peaks. 1,2-Xyl*p*: *m/z* 117, 1,4‑Xyl*p*: *m/z* 118.

**Table 4:** Composition (mol%) of liberated arabinan oligosaccharides after incubation of insoluble dietary fiber (IDF) of enzymatically treated apple pomace (raw and extruded) with endo-arabinanase (mean value ± range/2, *n* = 2). S1: only transport elements, S2: two reverse elements, S4: two reverse elements and a kneading block, bdl: below detection limit, nd: not detected.

Compound	Raw Material	S1	S2	S4
A-2a	84.1 ± 0.4	86.5 ± 0.7	84.6 ± 0.4	87.1 ± 1.8
A-4a	6.8 ± 0.1	5.8 ± 0.04	7.4 ± 0.1	7.1 ± 1.1
A-4b	1.6 ± 0.003	1.0 ± 0.1	1.7 ± 0.04	1.8 ± 0.3
A-5a	^bdl^	0.7 ± 0.04	0.6 ± 0.1	0.8 ± 0.2
A-5b	4.2 ± 0.3	3.3 ± 0.4	2.8 ± 0.2	1.8 ± 0.2
A-5c	2.3 ± 0.002	1.5 ± 0.1	1.4 ± 0.1	^bdl^
A-6a	1.0 ± 0.1	0.9 ± 0.03	1.2 ± 0.1	1.4 ± 0.3
A-7a	^nd^	0.1 ± 0.03	0.1 ± 0.004	^nd^
A-7b	^bdl^	0.1 ± 0.02	0.2 ± 0.001	^nd^

**Table 5:** Monosaccharide composition (mol%) of soluble dietary fiber (SDF) of enzymatically treated apple pomace (raw and extruded) after methanolysis (mean value ± range/2, *n* = 2) Fuc: fucose, Rha: rhamnose, Ara: arabinose, Gal: galactose, Glc: glucose, Xyl: xylose, Man: mannose, GalA: galacturonic acid, GlcA: glucuronic acid, S1: only transport elements, S2: two reverse elements, S4: two reverse elements and a kneading block, bdl: below detection limit.

	Raw Material	S1	S2	S4
Fuc	^bdl^	^bdl^	^bdl^	^bdl^
Rha	4.4 ± 0.1	5.4 ± 0.2	5.1 ± 0.2	5.6 ± 0.2
Ara	39.9 ± 0.04	36.3 ± 0.5	38.1 ± 0.01	41.2 ± 0.3
Gal	10.3 ± 0.2	12.1 ± 0.3	12.1 ± 0.2	13.7 ± 0.1
Glc	3.4 ± 0.2	4.4 ± 0.9	4.8 ± 0.4	3.4 ± 0.2
Xyl	3.4 ± 0.1	4.4 ± 0.4	4.7 ± 0.3	5.1 ± 0.2
Man	5.8 ± 0.01	5.4 ± 0.1	5.1 ± 0.04	4.4 ± 0.1
GalA	32.8 ± 0.2	32.0 ± 0.1	30.1 ± 0.4	26.7 ± 0.5
GlcA	^bdl^	^bdl^	^bdl^	^bdl^

## Data Availability

Not applicable.

## Appendix A

Protein content. The protein content was analyzed via liberated ammonia after Kjeldahl digestion of 100 mg of sample [21]. An ammonia electrode was used for ammonia detection.

Ash content. The ash content was determined gravimetrically after incineration (500 °C, 4 h).

## Appendix B

**Table A1:** Protein and ash contents (g/100 g dm, mean value ± standard deviation, *n* = 3) of extruded enzymatically treated apple pomace (raw and extruded). S1: only transport elements, S2: two reverse elements, S4: two reverse elements and a kneading block.

	Raw Material	S1	S2	S4
Protein content ^a^	2.6 ± 0.4 ^A^	2.3 ± 0.1 ^A^	2.8 ± 0.1 ^A^	2.5 ± 0.3 ^A^
Ash content ^a^	1.6 ± 0.03 ^A^	1.5 ± 0.02 ^A^	1.6 ± 0.06 ^A^	1.6 ± 0.1 ^A^

*^a^* Mean values within a row that are marked with different letters differ significantly (*p* < 0.05).

**Table A2:** Contents of free mono- and disaccharides (g/100 g dm, mean value ± standard deviation, *n* = 3) of enzymatically treated apple pomace (raw and extruded). S1: only transport elements, S2: two reverse elements, S4: two reverse elements and a kneading block.

	Raw Material	S1	S2	S4
Glucose ^a^	6.2 ± 0.9 ^A^	6.2 ± 1.3 ^A^	5.8 ± 0.2 ^A^	7.1 ± 1.9 ^A^
Fructose ^a^	12.7 ± 1.1 ^A^	11.7 ± 0.9 ^A^	11.8 ± 0.4 ^A^	12.6 ± 0.7 ^A^
Sucrose ^a^	4.1 ± 2.0 ^A^	3.4 ± 1.5 ^A^	4.0 ± 0.3 ^A^	3.4 ± 0.7 ^A^
Maltose ^a^	0.4 ± 0.1 ^A^	0.5 ± 0.1 ^A^	0.5 ± 0.1 ^A^	0.5 ± 0.1 ^A^

*^a^* Mean values within a row that are marked with different letters differ significantly (*p* < 0.05).

**Table A3:** Starch contents (g/100 g dm, mean value ± standard deviation, *n* = 3) of enzymatically treated apple pomace (raw and extruded). S1: only transport elements, S2: two reverse elements, S4: two reverse elements and a kneading block.

	Raw Material	S1	S2	S4
Starch content ^a^	8.3 ± 0.4 ^A^	7.8 ± 0.6 ^A^	7.7 ± 0.3 ^A^	8.0 ± 0.3 ^A^

*^a^* Mean values within a row that are marked with different letters differ significantly (*p* < 0.05).

**Table A4:** Composition (mol%) of galactan oligosaccharides after incubation of insoluble dietary fiber (IDF) and soluble dietary fiber (SDF) of enzymatically treated apple pomace (raw and extruded) with endo‑galactanase (mean value ± range/2, *n* = 2). S1: only transport elements, S2: two reverse elements, S4: two reverse elements and a kneading block, bdl: below detection limit, nd: not detected.

Compound	Raw Material		S1		S2		S4	
	IDF	SDF	IDF	SDF	IDF	SDF	IDF	SDF
G-2a	95.3 ± 0.7	100.0	93.3 ± 0.4	100.0	94.3 ± 0.2	100.0	93.7 ± 0.7	100.0
G-2b	^bdl^	^bdl^	4.2 ± 0.4	^bdl^	3.7 ± 0.1	^bdl^	2.5 ± 0.3	^bdl^
G-2c	4.7 ± 0.7	^nd^	2.4 ± 0.04	^nd^	1.9 ± 0.2	^nd^	2.5 ± 0.5	^nd^

**Table A5:** Glyosidic linkages of soluble dietary fiber (SDF) polysaccharides of enzymatically treated apple pomace (raw and extruded) (mol%, mean value ± range/2, *n* = 2). t: terminal, p: pyranose, f: furanose, Rha: rhamnose, Ara: arabinose, Gal: galactose, Glc: glucose, Man: mannose, Xyl: xylose, S1: only transport elements, S2: two reverse elements, S4: two reverse elements and a kneading block, nd: not detected.

	Raw Material	S1	S2	S4
1,2-Rha*p*	1.1 ± 0.02	1.4 ± 0.2	0.9 ± 0.04	2.1 ± 0.2
1,2,4-Rha*p*	0.4 ± 0.1	0.4 ± 0.1	0.4 ± 0.01	1.3 ± 0.5
∑ Rha	1.6 ± 0.1	1.8 ± 0.3	1.3 ± 0.1	3.4 ± 0.6
t-Ara*f*	17.5 ± 0.9	24.8 ± 1.2	23.6 ± 0.2	22.6 ± 0.6
t-Ara*p*	0.9 ± 0.2	1.0 ± 0.2	1.1 ± 0.2	0.7 ± 0.1
1,2-Ara*f*	0.7 ± 0.05	0.5 ± 0.01	0.5 ± 0.02	0.5 ± 0.001
1,3-Ara*f*	3.4 ± 0.1	3.8 ± 0.9	3.0 ± 0.01	3.4 ± 0.1
1,5-Ara*f*/1,4-Ara*p*	23.0 ± 0.5	22.2 ± 0.2	24.5 ± 0.4	22.6 ± 0.04
1,2,5-Ara*f*	2.8 ± 0.3	1.4 ± 0.7	1.3 ± 0.8	2.4 ± 0.04
1,3,5-Ara*f*	9.6 ± 0.6	8.1 ± 0.2	8.7 ± 0.1	9.0 ± 0.5
1,2,3,5-Ara*f*	10.9 ± 1.9	8.8 ± 0.7	9.1 ± 2.1	7.0 ± 0.03
∑ Ara	68.7 ± 4.6	70.8 ± 4.1	71.9 ± 3.8	67.9 ± 1.5
t-Gal*p*	2.6 ± 0.4	3.3 ± 0.4	2.0 ± 0.2	3.3 ± 0.2
1,4-Gal*p*	6.6 ± 0.4	3.7 ± 0.1	3.7 ± 0.3	4.6 ± 0.1
1,4,6-Gal*p*	2.0 ± 0.4	2.3 ± 0.2	2.0 ± 0.2	1.9 ± 0.1
∑ Gal	11.2 ± 1.2	9.3 ± 0.6	7.7 ± 0.7	9.8 ± 0.4
t-Glc*p*	1.0 ± 0.01	1.9 ± 0.8	0.5 ± 0.03	0.9 ± 0.1
1,4-Glc*p*	7.0 ± 1.7	4.3 ± 2.3	5.8 ± 0.7	4.9 ± 0.3
1,4,6-Glc*p*	1.4 ± 0.01	2.3 ± 1.0	2.6 ± 0.2	2.7 ± 0.2
∑ Glc	9.4 ± 1.7	8.6 ± 4.1	8.9 ± 1.0	8.5 ± 0.6
t-Man*p*	1.4 ± 0.2	^nd^	0.7 ± 0.1	1.0 ± 0.3
1,4-Man*p*	1.3 ± 0.2	0.8 ± 0.1	1.5 ± 0.4	1.2 ± 0.1
∑ Man	2.7 ± 0.4	0.8 ± 0.1	2.2 ± 0.5	2.2 ± 0.4
t-Xyl*p*	4.0 ± 0.5	6.3 ± 0.4	5.5 ± 0.2	5.8 ± 0.5
1,2-Xyl*p*^a^	1.9 ± 0.2	1.5 ± 0.05	1.5 ± 0.1	1.5 ± 0.01
1,4-Xyl*p*^a^	0.5 ± 0.5	1.0 ± 0.1	1.0 ± 0.01	0.9 ± 0.05
∑ Xyl	6.4 ± 1.3	8.8 ± 0.5	8.0 ± 0.3	8.1 ± 0.6

^a^ Coeluting, determined from the area ratio of the characteristic fragment ion peaks. 1,2-Xyl*p*: *m/z* 117, 1,4‑Xyl*p*: *m/z* 118.

**Table A6:** Composition (mol%) of arabinan oligosaccharides after incubation of soluble dietary fiber (SDF) of enzymatically treated apple pomace (raw and extruded) with endo-arabinanase (mean value ± range/2, *n* = 2). S1: only transport elements, S2: two reverse elements, S4: two reverse elements and a kneading block, bdl: below decetion limit, nd: not detected.

Compound	Raw Material	S1	S2	S4
A-2a	81.1 ± 2.6	85.0 ± 2.8	82.6 ± 2.4	80.5 ± 0.3
A-4a	10.4 ± 0.3	9.1 ± 0.5	9.7 ± 1.1	10.9 ± 0.1
A-4b	2.4 ± 0.6	1.5 ± 0.7	1.4 ± 0.5	1.8 ± 0.1
A-5a	^bdl^	1.1 ± 1.0	1.7 ± 0.8	2.1 ± 0.5
A-5b	4.0 ± 0.4	3.3 ± 0.7	4.5 ± 0.02	4.7 ± 0.2
A-5c	^bdl^	^bdl^	^bdl^	^bdl^
A-6a	^bdl^	^bdl^	^nd^	^nd^
A-7a	^nd^	^nd^	^nd^	^nd^
A-7b	2.0 ± 1.3	^nd^	^nd^	^nd^

**Table A7:** Monosaccharide composition (mol%) of low-molecular weight soluble dietary fiber (LMW-SDF) of enzymatically treated apple pomace (raw and extruded) after TFA hydrolysis (mean value ± standard deviation, *n* = 3) Rha: rhamnose, Ara: arabinose, Gal: galactose, Glc: glucose, Xyl: xylose, Man: mannose, GalA: galacturonic acid, S1: only transport elements, S2: two reverse elements, S4: two reverse elements and a kneading block, bdl: below detection limit.

	Raw Material	S1	S2	S4
Rha	^bdl^	^bdl^	^bdl^	^bdl^
Ara ^a^	56.6 ± 8.8 ^A^	59.7 ± 18.7 ^A^	66.9 ± 10.5 ^A^	72.2 ± 11.6 ^A^
Gal ^a^	4.4 ± 0.9 ^A^	^bdl^	^bdl^	3.8 ± 1.6 ^A^
Glc ^a^	21.9 ± 4.3 ^A^	15.1 ± 5.0 ^A,B^	12.3 ± 3.4 ^A,B^	10.0 ± 2.5 ^B^
Xyl	^bdl^	^bdl^	^bdl^	^bdl^
Man ^a^	3.7 ± 1.6 ^A^	6.8 ± 4.2 ^A^	9.3 ± 5.1 ^A^	3.0 ± 0.6 ^A^
GalA ^a^	13.4 ± 6.4 ^A^	18.4 ± 10.8 ^A^	11.6 ± 9.3 ^A^	11.0 ± 7.5 ^A^

*^a^* Mean values within a row that are marked with different letters differ significantly (*p* < 0.05).
